# PERCEPTIONS OF PERPETRATORS OF AGEISM

**DOI:** 10.1093/geroni/igz038.308

**Published:** 2019-11-08

**Authors:** Alison L Chasteen, Michelle Horhota, Jessica Crumley-Branyon

**Affiliations:** 1 University of Toronto, Toronto, Canada; 2 Furman University, Greenville, South Carolina, United States

## Abstract

What are the consequences for perpetrators who engage in different types of ageism? We compared young (n=316), middle-aged (n=464), and older adults’ (n=273) perceptions of a perpetrator who engaged in an ageist action. Participants read a vignette about a pedestrian (the perpetrator) offering unwanted help to an older woman crossing the street. We manipulated the ageism type (benevolent or hostile), the reaction of the older target (acceptance, moderate confrontation or strong confrontation) and assessed the overall impression of the perpetrator. Main effects emerged for Ageism Type and Age Group. Overall, participants rated the perpetrator more positively in the benevolent condition compared to the hostile condition. Middle-aged and older adults rated the perpetrator more positively than young adults did. A Time x Confront interaction suggested that the perpetrator’s overall impression was not impacted when the target of the ageist act accepted the action or moderately confronted the perpetrator. In contrast, when the target confronted the perpetrator strongly, the overall impression of the perpetrator decreased. An Ageism Type x Age Group x Time interaction on overall impression also emerged. There were no age differences when the perpetrator committed a hostile act of ageism. In contrast, in the benevolent condition young and older adults perceived the perpetrator more negatively after the target’s reaction, whereas middle-aged adults did not adjust their impression. Taken together, these results suggest that young and older adults may be less accepting of benevolent ageism compared to middle-aged adults.

